# The arginine methyltransferase PRMT1 regulates IGF-1 signaling in breast cancer

**DOI:** 10.1038/s41388-019-0694-9

**Published:** 2019-01-28

**Authors:** Ali Choucair, Thuy Ha Pham, Soleilmane Omarjee, Julien Jacquemetton, Loay Kassem, Olivier Trédan, Juliette Rambaud, Elisabetta Marangoni, Laura Corbo, Isabelle Treilleux, Muriel Le Romancer

**Affiliations:** 1 INSERM U1052, Centre de Recherche en Cancérologie de Lyon, Lyon, France; 2 CNRS UMR5286, Centre de Recherche en Cancérologie de Lyon, Lyon, France; 3 Université Lyon 1, Lyon, France; 4 Cancer Research UK, Cambridge Institute, University of Cambridge, Cambridge, CB2 0RE UK; 5 Clinical Oncology Department, Faculty of Medicine, Cairo University, Cairo, Egypt; 6 Oncology Department, Centre Leon Bérard, Lyon, France; 7 Translational Research Department, Institut Curie, 75005 Paris, France; 8 Pathology Department, Centre Leon Bérard, Lyon, France

**Keywords:** Cell signalling, Breast cancer

## Abstract

Aside from its well-known nuclear routes of signaling, estrogen also mediates its effects through cytoplasmic signaling. Estrogen signaling involves numerous posttranslational modifications of its receptor ERα, the best known being phosphorylation. Our research group previously showed that upon estrogen stimulation, ERα is methylated on residue R260 and forms the mERα/Src/PI3K complex, central to the rapid transduction of nongenomic estrogen signals. Regulation of ERα signaling via its phosphorylation by growth factors is well recognized, and we wondered whether they could also trigger ERα methylation (mERα). Here, we found that IGF-1 treatment of MCF-7 cells induced rapid ERα methylation by the arginine methyltransferase PRMT1 and triggered the binding of mERα to IGF-1R. Mechanistically, we showed that PRMT1 bound constitutively to IGF-1R and that PRMT1 became activated upon IGF-1 stimulation. Moreover, we found that expression or pharmacological inhibition of PRMT1 impaired mERα and IGF-1 signaling. Our findings were substantiated in a cohort of breast tumors in which IGF-1R expression was positively correlated with ERα/Src and ERα/PI3K expression, hallmarks of nongenomic estrogen signaling, reinforcing the link between IGF-1R and mERα. Altogether, these results provide a new insight into ERα and IGF-1R interference, and open novel perspectives for combining endocrine therapies with PRMT1 inhibitors in ERα-positive tumors.

## Introduction

Breast cancer is the second most common cancer affecting women worldwide after lung cancer. Although patients are often diagnosed in the early and curable stages, the treatment of metastatic breast cancer remains a major clinical challenge. Estrogen is frequently associated with breast cancer development as 80% of breast cancers express its receptor, ERα. ERα-positive patients are treated with hormonotherapy, though acquired resistance to hormonal treatments has emerged, highlighting the need for novel strategies to improve clinical outcome [1]. ERα signaling is quite complex and involves many actors, not only from its typical genomic/nuclear pathway but also from its nongenomic pathway [2, 3], although only the nuclear ERα status is currently taken into account in the decision-making process associated with treatment management. Yet, the nongenomic pathway has been extensively described. In detail, estrogen induces the interaction of ERα with Src, PI3K and other proteins to form a large complex that activates downstream proliferative signaling pathways such as MAPK and PI3K/Akt [2, 3]. Furthermore, our research group demonstrated that upon estrogen stimulation, ERα is methylated by the arginine methyltransferase PRMT1 on the R260 residue located at the junction between the DNA binding domain (DBD) and the hinge region. We provided evidence that this event is a prerequisite for the formation of the mERα/Src/PI3K complex and for the activation of downstream signaling [4]. We also showed that this pathway is activated in aggressive human breast tumors and could constitute a new prognostic marker [5].

In the search for novel ERα targets, upstream events leading to the regulation of ERα through estrogen-independent pathways have also been investigated and were associated with kinases that are activated by growth factor receptors, such as the epidermal growth factor receptor (EGFR) and the insulin-like growth factor 1 receptor (IGF-1R) [6, 7]. IGF-1, for instance, activates the transcriptional activity of ERα by phosphorylating its Ser167 residue through the Akt/mTOR/S6K1 axis [8].

Having previously reported the importance of ERα methylation and of its interactions with downstream nongenomic signaling factors, we wondered whether ERα methylation could also been induced independently of estrogen. We revealed that, similarly to E_2_, IGF-1 regulates PRMT1-induced ERα methylation. Indeed, we showed that the binding of PRMT1 to IGF-1R is a prerequisite for IGF-1 signaling probably via the regulation of ERα methylation. Interestingly, IGF-1R also phosphorylates ERα on residue Y219, and this interaction may be important for downstream signaling events. Finally, our data provide a rationale for the use of PRMT1 inhibitors to concomitantly target IGF-1 and estrogen nongenomic pathways in ERα-positive breast cancer therapies.

## Results

### The growth factor IGF-1 induces ERα methylation

**Fig. 1:**
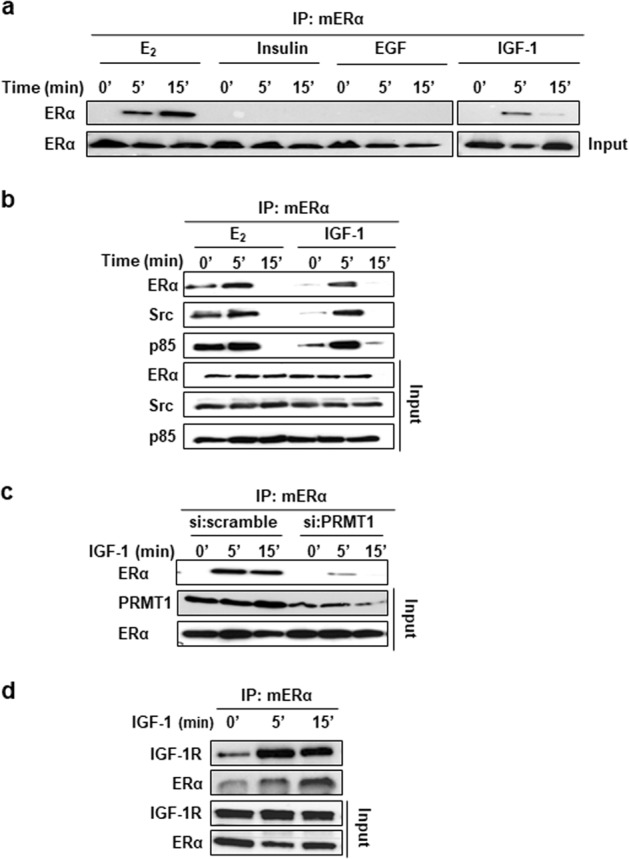
IGF-1 triggers ERα methylation. **a** MCF-7 cells grown in serum-free medium were treated with E_2_ (10^–8^ M), insulin (100 ng/ml), EGF (100 ng/ml) or IGF-1 (40 ng/ml) for the indicated times. ERα methylation was then assessed by performing immunoprecipitation assays with the anti-mERα antibody followed by western blotting with an ERα antibody. ERα input is also shown. **b** MCF-7 cells were treated with E_2_ or IGF-1 as in (**a**), and then tested for ERα methylation. The immunoprecipitates were blotted with anti-ERα, anti-Src, and anti-p85 (PI3K antibody). The amount of ERα, p85, and Src in the different samples was determined by western blotting. **c** Lysates of MCF-7 cells transfected with control siRNA duplexes or siRNAs targeting PRMT1 were tested for IGF-1-induced ERα methylation as in (**a**). Expression of PRMT1 and ERα was checked by western blotting. **d** MCF-7 cells were treated with IGF-1 for the indicated times. mERα was then immunoprecipitated with the specific antibody followed by western blotting with anti-ERα and IGF-1R antibodies. The expression of ERα and IGF-1R in the inputs was evaluated by western blotting using the corresponding antibodies. IGF-1 insulin-like growth factor 1, EGF epidermal growth factor

To investigate whether other stimuli, such as growth factors shown to regulate ERα phosphorylation, could trigger mERα, we treated MCF-7 cells with insulin, EGF or IGF-1 (E_2_ being our internal positive control) for different periods of time before conducting immunoprecipitation assays using an antibody specifically recognizing di-methylated ERα on R260, as previously described [4]. Among the ligands tested, IGF-1 alone triggered ERα methylation in a rapid and transitory manner reminiscent of the effect produced by E_2_ treatment (Fig. 1a). Moreover, we observed that IGF-1 stimulated the interaction of mERα with several proteins of its regulatory complex, namely Src and p85 (regulatory subunit of PI3K) (Fig. 1b), indicating that IGF-1 may have a similar modulatory activity on mERα to E_2_. However, methylation kinetics were more rapid and confirmed the results previously published showing that this process could vary according to experimental conditions but was always rapid and transient [4, 9, 10]. Next, by conducting siRNA experiments targeting PRMT1, the enzyme directly responsible for ERα methylation, we observed that PRMT1 knockdown strongly reduced IGF-1-induced ERα methylation (Fig. 1c), confirming its concomitant implication in E_2_- and IGF-1-induced mERα. Moreover, IGF-1 stimulation also fostered the interaction between mERα and IGF-1R (Fig. 1d), indicating that IGF-1R may also be implicated in the IGF-1/mERα signaling pathway, corroborating a previous study which reported that treatment of cells with IGF-1 induces a partial relocalization of ERα into the cytoplasm [11].

Altogether, these results show that IGF-1 triggers PRMT1-induced ERα methylation, and the recruitment of Src, PI3K and IGF-1R, suggesting a new mechanism of IGF-1 signaling pathway regulation.

### IGF-1R interacts with PRMT1 and regulates its activity

**Fig. 2:**
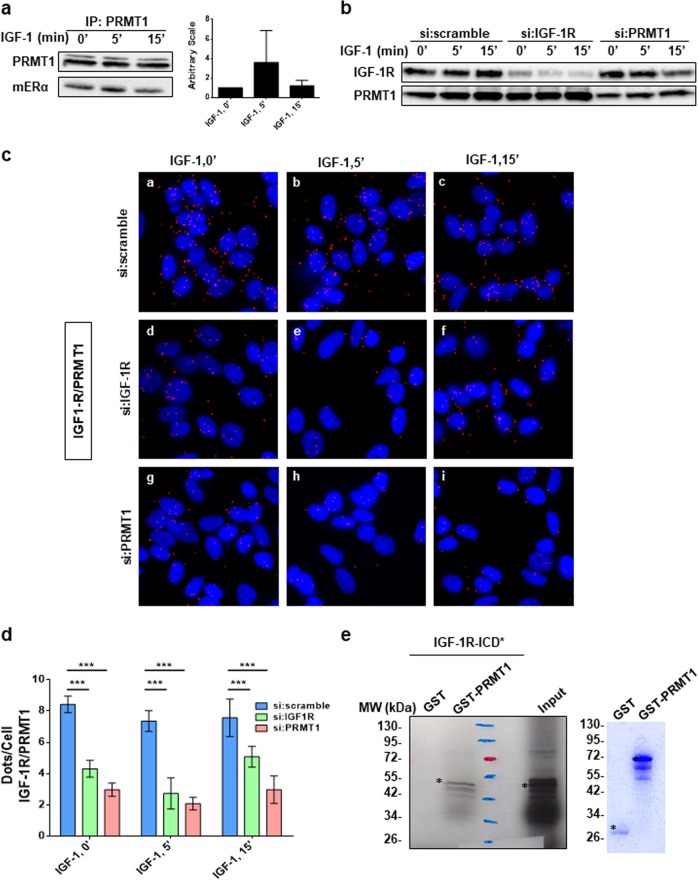
IGF-1R interacts with PRMT1. **a** MCF-7 cells were treated with IGF-1 for the indicated times, cell lysates were then immunoprecipitated with anti-PRMT1 antibody and its enzymatic activity was evaluated by performing an in vitro methylation assay using the GST-hinge of ERα as a substrate, detected by western blot using the anti-mERα antibody. Quantification of the signal was performed by computer-assisted analysis (right-hand panel). This result is representative of two independent experiments. **b** MCF-7 cells were transfected with si:scramble or siRNAs targeting IGF-1R or PRMT1 for 72 h, then treated with IGF-1 for different times. The efficacy of protein inhibition was verified by western blot using the corresponding antibodies. **c** After siRNA transfection and fixation, proximity ligation assay experiments were performed to evaluate IGF-1R/PRMT1 interaction using IGF-1R- and PRMT1-specific antibodies. The detected dimers are represented by red dots. The nuclei were counterstained with mounting medium containing DAPI (blue) (Obj: ×60). **d** Quantification of the number of dots per cell was performed by computer-assisted analysis as reported in the Materials and Methods section. The mean ± s.e.m. of one experiment representative of three experiments is shown. The *P* value was determined using the Student *t* test. ***P < 0.001. **e** Radioactive GST pull-down assay was performed by incubating the in vitro ^35^S-labeled intracellular domain of IGF-1R (IGF-1R-ICD*) with GST and GST-PRMT1. The corresponding Coomassie-stained gel is shown in the right-hand panel. *Indicates the full-length fusion proteins. IGF-1R insulin-like growth factor 1 receptor

Having shown that PRMT1 methylated ERα in the presence of IGF-1, we wondered whether IGF-1 itself could regulate its enzymatic activity. Since no specific markers have been available for measuring endogenous PRMT1 activity, we conducted immunoprecipitation assays using an antibody specifically recognizing PRMT1 (Supplementary Fig. S1) extracted from fresh cells and measured its activity via an in vitro methylation experiment, as previously described for measuring kinase activities [12]. For this purpose, MCF-7 cells were treated with IGF-1 for different periods of time prior to PRMT1 immunoprecipitation. Its enzymatic activity was then tested using the hinge fragment of ERα containing the R260 residue as an exogenous substrate. We found that the level of methylation increased after 5 min of exposure to IGF-1, and then decreased at 15 min (Fig. 2a), showing that PRMT1 activity is increased upon IGF-1 treatment.

Furthermore, after performing proximity ligation assays (PLA), using cells knocked down either for IGF-1R or PRMT1 (efficacy of the siRNA knockdown is shown in Fig. 2b), to verify their specific interaction, we clearly observed that IGF-1R interacted with PRMT1 (Fig. 2c and Supplementary Fig. S2,a). This interaction, as indicated by the presence of red dots (Fig. 2c and Supplementary Fig. S2,a) and their subsequent quantification (Fig. 2d), was cytoplasmic and independent of IGF-1. The signals strongly diminished in MCF-7 cells, in which the expression of either IGF-1R (panels d, e, f) or PRMT1 (panels g, h, i) was knocked down, demonstrating the specificity of the signal that was further confirmed by a coimmunoprecipitation approach (Supplementary Fig. S2,b). Since the two proteins coimmunoprecipitated, we investigated whether they interacted more directly by conducting a GST pull-down approach, and we found that the intracellular domain of IGF-1R (ICD) interacts specifically with GST-PRMT1 (Fig. 2e).

In conclusion, our results showed a constitutive interaction between IGF-1R and PRMT1, suggesting a role for PRMT1 in IGF-1 signaling.

### PRMT1 influences IGF-1 signaling

**Fig. 3:**
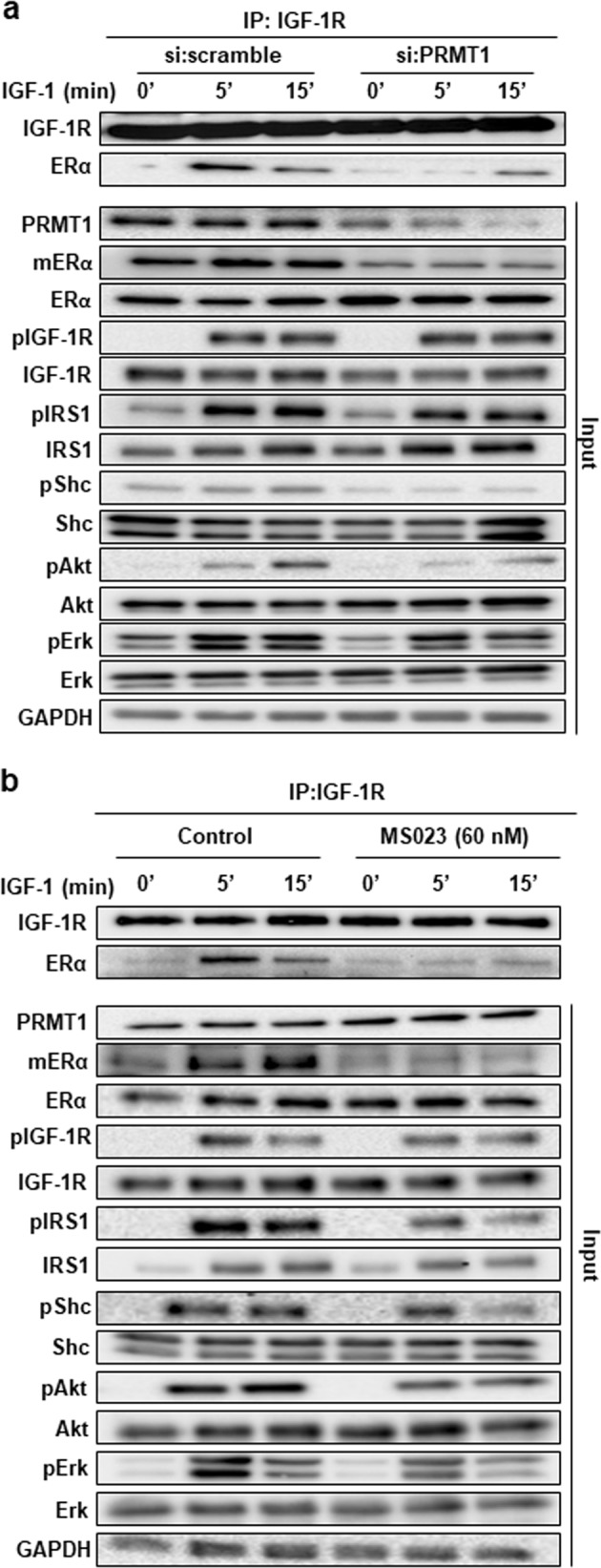
PRMT1 influences IGF-1 signaling. **a** MCF-7 cells were transfected with si:scramble or a pool of siRNAs targeting PRMT1 for 72 h, and then treated with IGF-1 for different times. Cell lysates were subsequently coimmunoprecipitated with the anti-IGF-1R antibody and detected by western blot analysis for the presence of ERα and IGF-1R, using the corresponding antibodies. The expression of mERα, ERα, PRMT1 and proteins involved in IGF-1 signaling was also evaluated by western blot using the corresponding antibodies. GAPDH expression was also assessed as a loading control. **b** MCF-7 cells were treated with the PRMT1 inhibitor (60 nM) 48 h before IGF-1 treatment, cell lysates were immunoprecipitated with anti-IGF-1R antibody and detected by western blot for the presence of IGF-1R and ERα, downstream IGF-1 signaling was then studied by western blot using the corresponding antibodies as in Fig. 3a. GAPDH expression was also assessed as a loading control. IGF-1 R insulin-like growth factor 1 receptor

Having shown that PRMT1 interacts with IGF-1R, we investigated the role of PRMT1 in IGF-1/IGF-1R signaling. Previous studies have shown that activation of the IGF-1R signaling pathway promotes proliferation, survival, and metastasis of breast cancer cells [13]. IGF-1R, when activated by ligand binding, is auto phosphorylated on tyrosine residues such as Y1135 in the kinase domain, thus activating adaptor proteins namely Src homology, the collagen domain protein (Shc) and the insulin receptor substrate 1 (IRS1) [14–16]. IGF-1R then triggers the proliferative signaling via two main pathways, ERK1/2 and PI3K/Akt respectively through Shc and IRS1 [13, 17]. Consistently, we knocked down PRMT1 in MCF-7 cells and studied the activation of these two downstream IGF-1R signaling events. For this and further experiments, we used a new batch of mERα antibody that recognizes the endogenously modified protein by western blot, thus excluding the need for immunoprecipitation assays (Supplementary Fig. S3). Though PRMT1 inhibition did not modify the basal levels of the proteins tested (Fig. 3a), it clearly decreased IGF-1-induced mERα, p-Shc (Y239/240), p-IRS1 (Y608/612) and their downstream p-Akt and p-ERK. These data were then validated using a new specific PRMT1 inhibitor, MS023 [18], which we initially tested (at different doses) on a known substrate of PRMT1, namely dimeR3Histone H4 (Supplementary Fig. S4,a) [19], as well as on mERα. An appropriate dose of 60 nM inhibited ERα methylation upon IGF-1 treatment (Supplementary Fig. S4,b, c) and was thus further used in coimmunoprecipitation assays (Fig. 3b). Similarly to si:PRMT1, MS023 also inhibited IGF-1 signaling, as evidenced by a significant decrease in p-IRS1, p-Shc, p-Akt, and p-ERK (Fig. 3b). In parallel, we unveiled that inhibiting PRMT1 activity did not impair IGF-1R auto-phosphorylation on residue Y1135, indicating that the regulation occurs downstream of this event. In addition, to assess whether PRMT1 could regulate IGF-1 signaling by regulating IGF-1R internalization, we studied IGF-1R localization by immunofluorescence upon MS203 treatment and found no modification (Supplementary Fig. S5).

### IGF-1R interacts directly with ERα

**Fig. 4:**
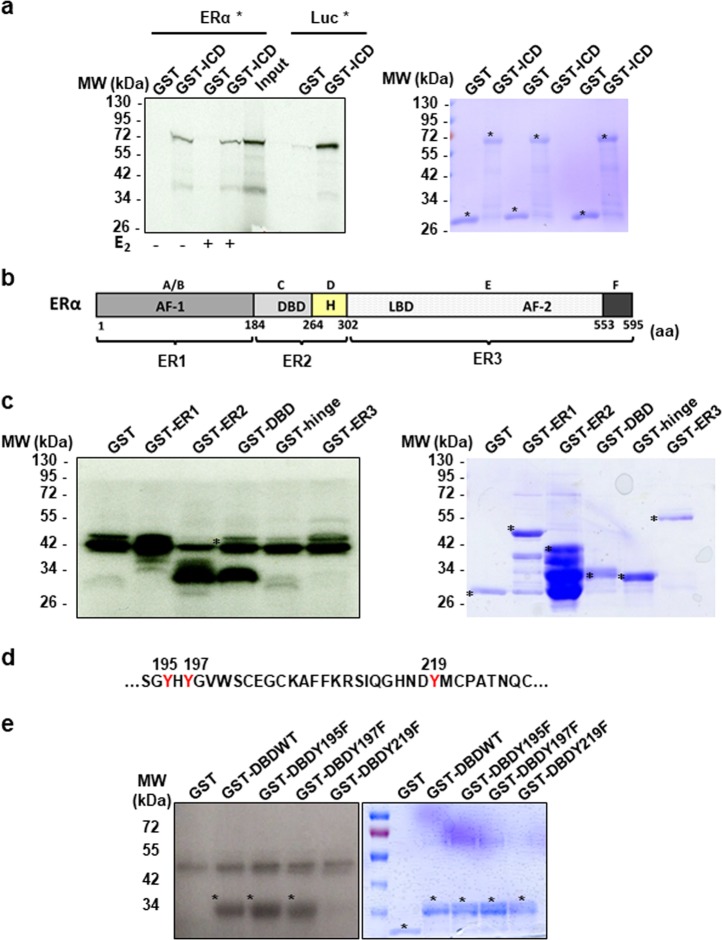
IGF-1R interacts with ERα and triggers its phosphorylation. **a** A radioactive GST pull-down assay was performed by incubating labeled in vitro ^35^S-labeled ERα or luciferase as a negative control with GST and GST-IGF-1R/ICD in the presence or absence of E_2_ (10^–6^ M). The corresponding Coomassie-stained gel is shown in the right-hand panel. *Indicates the fusion proteins. **b** ERα is divided into functional domains. ER1 is composed of the activation Function-1 (AF-1), ER2 contains the DNA binding domain (DBD) along with the hinge domain, and ER3 contains the ligand-binding domain (LBD) and the activation Function-2 (AF-2). **c** In vitro phosphorylation experiments were performed by incubating active IGF-1R with [^32^Pγ] ATP and GST or ERα fragments fused to GST (ER1, ER2, DBD, hinge, ER3). The phosphorylated proteins were visualized by autoradiography (left-hand panel). The corresponding Coomassie-stained gel is shown in the right-hand panel. *Indicates the full-length fusion proteins. **d** The amino acid sequence of a region of the DBD of ERα is shown and the three tyrosine residues are highlighted in red. **e** GST, GST-DBD WT or mutant Y195F, Y197F and Y219F were used as substrates for IGF-1R phosphorylation (left panel). The corresponding Coomassie-stained gel is shown in the right-hand panel. *Indicates the full-length fusion proteins. IGF-1R insulin-like growth factor 1 receptor

These coimmunoprecipitation experiments also revealed that PRMT1 was required for the interaction between IGF-1R and ERα. Indeed, siRNA knockdown and MS023 inhibition of PRMT1 both impaired ERα and IGF-1R coimmunoprecipitation (Fig. 3a, b), which was further confirmed by PLA (Supplementary Fig. S6 for validation of set-up and Supplementary Fig. S7). We then attempted to better understand the mechanisms underlying this interaction. With the finding that ERα may bind IGF-1R at the plasma membrane via the adaptor IRS1 [20], we studied the involvement of IRS1 in IGF-1-induced ERα methylation, but revealed that IRS1 knockdown via siRNA had no effect on mERα (Supplementary Fig. S8). Based on this result, we hypothesized that ERα could bind directly to IGF-1R. This direct interaction was examined by a GST pull-down approach, which revealed that radioactive ERα interacts specifically with the ICD of IGF-1R independently of the presence of E_2_ (Fig. 4a), and more precisely at the level of the D2 domain that contains the kinase activity of IGF-1R (Supplementary Fig. S9). In line with these results, we wondered whether ERα could be a substrate for IGF-1R and performed an in vitro phosphorylation assay using the active IGF-1R in the presence of purified fragments of ERα fused to GST (Fig. 4b). The results demonstrated that the fragment containing the DBD was the only one to be phosphorylated (Fig. 4c). Moreover, within the DBD sequence, three tyrosine residues, Y195, Y197 and Y219, were observed (Fig. 4d). The point-substitution of these tyrosine residues by phenylalanine residues had little effect on IGF-1R-induced phosphorylation, except in the case of the Y219 substitution (Fig. 4e). This observation led to another hypothesis that Y219 residue could play a role in the IGF-1R/ERα interaction. We transfected MCF-7 cells with empty pSG5-Flag, pSG5-FlagERα wild-type or the mutant Y219F (Supplementary Fig. S10,a) then studied the interaction by PLA using the anti-Flag and the anti-IGF-1R antibodies. According to the PLA results, the interaction was detectable only in the cells overexpressing wild-type ERα, while in cells transfected with the mutant Y219F ERα, the interaction significantly decreased (Supplementary Fig. S10,b, c).

### Crosstalk between IGF-1R and ERα in breast tumors

**Fig. 5:**
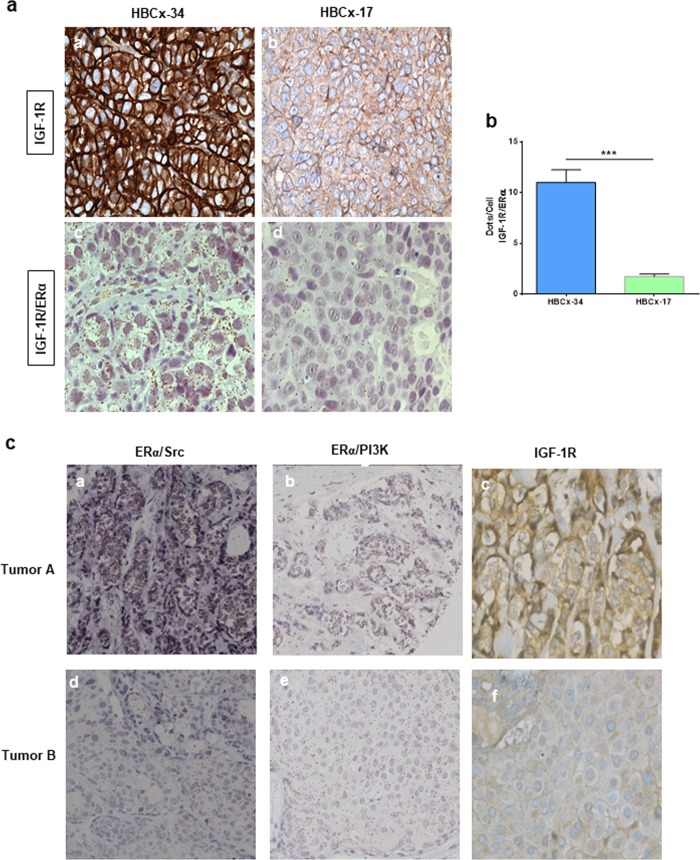
Crosstalk between IGF-1R and ERα in breast tumors. **a** Tumors from PDX models of breast cancer were embedded in paraffin. IGF-1R expression was assessed by IHC staining (panels a and b). A bright field PLA was performed to study ERα/IGF-1R interaction in the two PDX models (panels c and d). The brown dots represent protein−protein interactions (×40 magnification). **b** The interactions were quantified as described in the Materials and Methods section. The *P* value was determined using the Student’s *t* test. ***P < 0.001. **c** For each tumor, we analyzed by PLA the levels of ERα/Src (panels a, d), ERα/PI3K (panels b, e) along with IGF-1R expression by immunohistochemistry (panels c, f). IGF-1R insulin-like growth factor 1 receptor, PDX patient-derived xenograft, PLA proximity ligation assays

Having unveiled this interaction in vitro, we then validated our data in vivo. To achieve this, we analyzed the IGF-1R/ERα interaction in breast tumors by bright field PLA, in which the presence of protein interactions is visualized as brown dots. We detected interactions in the cytoplasm of a patient-derived breast xenograft (PDX) of ERα+ breast cancer (Fig. 5a, panel c) that strongly expressed IGF-1R at the plasma membrane and in the cytoplasm (Fig. 5a, panel a), and quantified these (Fig. 5b). Unlike HBCx-34, the ERα-negative PDX (HBCx-17) did not express IGF-1R (Fig. 5a, panel b) and displayed very few interactions (Fig. 5a, panel d and 5b). These results confirmed that IGF-1R interacts with ERα in the cytoplasm of human breast tumors. To substantiate these findings, we then studied the association of IGF-1R expression (by immunohistochemistry (IHC) in 440 breast tumor specimen) with ERα/Src and ERα/PI3K expression, two markers already shown to be strongly correlated with nongenomic mERα signaling in a previous study [5]. Representative images of a tumor expressing high levels of ERα/Src, ERα/PI3K and IGF-1R and one expressing low levels of the three markers are shown in Fig. 5c. Statistical analyses revealed that the expression of ERα/Src and ERα/PI3K was positively correlated with IGF-1R expression (Table 1), suggesting that IGF-1R activation may also trigger the formation of the complex containing mERα/Src/PI3K in vivo as shown in MCF-7 cells (Fig. 1b).

**Table 1:** Correlation between IGF-1R expression (by IHC) and ERα/Src or ERα/PI3K interactions (by PLA) using Fisher’s exact test

Variable		IGF-1R low (*H* ≤ 100)		IGF-1R high (*H* > 100)		*P* value
		No. 202	(%) (50%)	No. 202	(%) (50%)	
ERα/SRC	Low (≤10)	132	(63.8)	106	(54.1)	0.048
	High (>10)	75	(36.2)	90	(45.9)	
ERα/PI3K	Low (≤9)	136	(67.3)	106	(52.5)	0.014
	High (>9)	66	(32.7)	96	(47.5)	

The level of ERα/Src and ERα/PI3K interactions is positively associated with higher IGF-1R expression in breast tumor samples

## Discussion

Approximately 80% of breast cancers express ERα and endocrine therapies have led to significant improvements in patient survival. However, their efficacy is limited by intrinsic and acquired therapeutic resistance. Among the causes of resistance, receptor tyrosine kinase signaling, namely through IGF-1R, has for instance been associated with tamoxifen resistance [21], which is likely due to the bidirectional crosstalk between ERα and receptor tyrosine kinase signaling. In the present study, we shed light on a novel interaction between ERα and IGF-1R involving the enzymatic methylation activity of PRMT1. Abundant studies have highlighted a crosstalk between IGF-1R and ERα in breast tumor cells. Indeed, the dual treatment of cells with estrogen and IGF-1 results in greater proliferation than exposure to either ligand individually [22, 23]. Moreover, exposure to the carcinogen 7,12-dimethylbenz(a)anthracene in dwarf rats that exhibit low levels of circulating IGF-1 produces fewer ERα-positive breast tumors than in normal rats [24]. The potential synergy between ERα and IGF-1R is underscored by studies showing enhanced antitumor efficacy upon combining antiestrogen agents with IGF-1R inhibitors [25, 26]. At the molecular level, it has been clearly demonstrated that the crosstalk between ERα and IGF-1 is bidirectional as ERα regulates the IGF-1 pathway, while IGF-1 activates ERα in a ligand-independent manner. Indeed, IGF-1 induces ERα expression, phosphorylates it as well as its coactivators, initializing its transcriptional activity [6, 27]. Conversely, estrogen influences the IGF-1 pathway by increasing the expression of both IGF-1R and IRS1 in breast cancer cells [28]. Moreover, ERα was shown to regulate the degradation of the IRS1 in breast cancer cells [29].

In this study, we found that, similarly to E_2_, IGF-1 triggered ERα methylation in MCF-7 cells. This event is not common to growth factors as insulin and EGF were not involved in this posttranslational modification. The time course of IGF-1 also follows that of E_2_, as ERα methylation is rapidly induced and transitory, suggesting the removal of the methylation mark. Based on previous findings from our research group, we hypothesize that this decrease in methylation could involve the arginine demethylase JMJD6, which has been proven to demethylate mERα and disrupt the complex containing mERα/Src/PI3K [10].

In a physiological context, IGF-1R is a tyrosine kinase cell surface receptor which participates in the regulation of cell growth and metabolism [30]. However, increased expression of IGF-1R and/or IGF-1 is associated with various types of cancers, notably in breast cancer, in which breast cancer cells often coexpress IGF-1R and ERα [31, 32]. IGF-1R has also been shown to be upregulated in tamoxifen-resistant breast cancer cells [17, 33] and to participate in antiestrogen resistance [34]. IGF-1R expression has different prognostic values for patients with breast cancers of different molecular subtypes. Indeed, in hormone-receptor-positive breast cancers, it was correlated with a better survival, although in triple-negative breast cancer, it predicted poor survival [32, 35]. So far, monotherapies targeting IGF-1 signaling have largely been disappointing and success has been limited by the lack of validated predictive biomarkers. In addition, due to their lack of specificity, IGF-1R tyrosine kinase inhibitors are associated with hyperglycemia because of interference with insulin signaling [17].

Among new therapeutic targets, PRMT1 appears to be a good candidate as it is involved in IGF-1R/ERα interaction. We can speculate that IGF-1R could interact with the methylated form of ERα, which we demonstrated to be exclusively expressed in the cytoplasm of breast tumors [4, 5]. Moreover, the analysis of IGF-1R expression in a cohort of breast cancer patients highlighted a strong correlation between IGF-1R and either ERα/Src or ERα/PI3K expression. In a previous cohort, we clearly showed that ERα/Src expression and ERα/PI3K expression are strongly correlated with mERα expression and the downstream activation of Akt [5]. Combining these two observations, we can speculate that IGF-1R and mERα expression might also be correlated, reinforcing our in vitro results. We demonstrated by different approaches that PRMT1 constitutively binds to IGF-1R, and PRMT1 becomes activated upon IGF-1 treatment, leading to ERα methylation. This result was striking as PRMT1 is mainly expressed in the nucleus [36], where it regulates transcription via histone methylation [19]. However, PRMT1 has already been shown to transit outside of the nucleus [37]. In addition, several articles mentioned PRMT1 binding to membrane receptors. In 1997, a two hybrid screen identified PRMT1 as a partner of the type I interferon receptor, independently of interferon [38]. A decade later, PRMT1 was shown to bind to and methylate the Igα subunit of the B-cell antigen receptor, to regulate B-cell differentiation [39]. Moreover, a few years ago, Xu et al. demonstrated that upon BMP4 treatment, PRMT1 is recruited to the BMP type II receptor, modulating downstream signaling [40]. The localization of PRMT1 at the level of the plasma membrane can also be explained by the identification of a spliced variant (e.g. PRMT1-V2), present at the membrane due to an NES insertion in the N-terminal part of the protein [41, 42].

Furthermore, we illustrated that PRMT1 plays a crucial role in IGF-1 signaling and its expression or pharmacological inhibition impairs downstream signaling, such as Akt and ERK phosphorylation. Under our experimental conditions, IGF-1R phosphorylation was not impacted, indicating that the regulation of the signaling pathway might occur after this event. We speculated that PRMT1 may regulate IGF-1 signaling by methylating IGF-1R, but we were unable to detect any methylation (data not shown). However, we only investigated the putative methylation on the intracellular domain of the receptor, and we cannot exclude that PRMT1 could methylate extracellular domains, as it was demonstrated for EGFR [43]. To explain the effect of PRMT1 on downstream IGF-1 signaling, we reasoned that a decrease in ERα recruitment to IGF-1R may impact the binding of the adaptors IRS1 and Shc, therefore impeding their phosphorylation by IGF-1R. Our findings corroborated those of Tian et al. who showed that ERα was essential for IGF-1-induced IRS1 phosphorylation [20]. Moreover, we demonstrated that ERα binds directly with the intracellular domain of IGF-1R, resulting in the phosphorylation of ERα on the Y219 residue, which is located in the DBD of ERα. Interestingly, the ERα Y219F mutant lost the capacity of binding to IGF-1R, suggesting that Y219 phosphorylation could stabilize the interaction. This tyrosine residue is phosphorylated by the kinase c-Abl and this phosphorylation has once been shown to regulate the transcriptional activity of ERα via the modulation of its binding to DNA [44]. These authors produced a glutamic acid mutant that mimics the phosphorylation, and led to an increase in cell proliferation and invasion. According to our results, the observed effects could be attributed to the activation of the IGF-1 signaling via the binding of IGF-1R to ERα. We cannot exclude that palmitoylation could also be involved in IGF-1 signaling. Indeed, this modification has been shown to be involved in the localization of ERα at the plasma membrane [45], as well as in the interaction between IGF-1R/ERα [46].

Even though our results suggest that PRMT1-induced ERα methylation is involved in IGF-1 signaling, we should not overlook the likelihood that other PRMT1 substrates could be involved. To address this issue in the near future, we plan to use genome editing to generate MCF-7 cell lines harboring a R260K *ESR1* mutation to decipher the precise role of mERα in IGF-1 signaling.

**Fig. 6:**
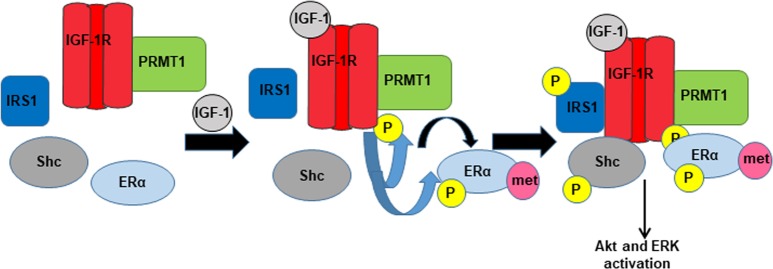
Model of IGF-1 signaling proposed in our study. IGF-1 insulin-like growth factor 1

Taking all of our findings together, we propose the model depicted in Fig. 6. In detail, IGF-1R constitutively binds to PRMT1 independently of its ligand. The presence of IGF-1 fosters PRMT1 activation, which in turn, methylates ERα. This event triggers its binding to IGF-1R and its phosphorylation on residue Y219, which stabilizes their interaction. Next, IGF-1R phosphorylates IRS1 and Shc on tyrosine residues, which form docking sites for PI3K and Grb2, activating Akt and ERK pathways, respectively [17]. Targeting PRMT1 could thus be a specific way of inhibiting IGF-1 signaling, since insulin does not trigger ERα methylation. Moreover, PRMT1 inhibitors could concomitantly target nongenomic ERα and IGF-1 signaling, two pathways largely implicated in breast cancer development.

## Materials and methods

### Cell culture and transfections

MCF-7 cells were maintained at 37 °C in Dulbecco’s modified Eagle’s medium supplemented with 10% fetal calf serum, 1% nonessential amino acids and 2% of penicillin/streptomycine. The cell line has been authenticated by Eurofins. Prior to treatment with ligands, cells were grown for 48 h in phenol red-free medium supplemented with 10% charcoal-stripped serum (Biowest), in order to remove steroid hormones or in serum-free medium for IGF-1 treatment. The cells were then treated for different times with E_2_ (Sigma) 10^–8^ M or IGF-1 (4×10^–5^ µg/µl) from Peprotech. When stated, cells were treated with the PRMT1 inhibitor MS023 (Tocris Bioscience).

For knockdown experiments, specific siRNAs or scramble siRNA (Eurogentec) (50 nM) were transfected into MCF-7 cells using the lipofectamine 2000 reagent (Invitrogen). The targeted sequences are given in Supplementary Table 1. After 72 h of transfection, proteins were analyzed.

For overexpression experiments, pSG5-Flag-tagged vectors were transfected into MCF-7 cells using Jetprime reagent (Ozyme) according to the manufacturer’s protocol. Thirty hours after transfection, cells were collected and analyzed.

### Antibodies

The dilutions and antibodies used for each method are listed in Supplementary Table 2.

### PDX tumors

We used tumors from human breast PDX provided by Dr. Marangoni of the Curie Institute, Paris. HBCx-17 and HBCx-34 had previously been established from early stage breast cancers and characterized [47, 48]. HBCx-17 expresses neither ERα nor IGF-1R, while HBCx-34 expresses both ERα and IGF-1R [47].

### Cloning and vectors

The vectors used and the cloning procedure are described in the Supplementary Material section.

### Immunoprecipitation and western blotting

Cells were lyzed using RIPA buffer (50 mM Tris HCl, pH 8, 150 mM NaCl, 1 mM ethylenediamine tetra-acetic acid (EDTA), 1% NP-40 and 0.25% deoxycholate) supplemented with protease inhibitor tablets (Roche Molecular Biochemicals) and phosphatase inhibitors (1 mM sodium fluoride, 1 mM Na_3_VO_4_ and 1 mM β-glycerophosphate). Protein extracts were incubated with primary antibodies overnight at 4 °C on a shaker. According to antibody species, either Protein G or A-Agarose beads were added, and the mixture was incubated for 2 h at 4 °C. The immunoprecipitated proteins were separated by sodium dodecyl sulfate-polyacrylamide gel electrophoresis (SDS-PAGE) and analyzed by western blot, then visualized by electrochemiluminescence (Roche Molecular Biochemicals).

### Proximity ligation assay (PLA)

This technology exposes protein/protein interactions in situ [49]. Briefly, cells were seeded and fixed with cold methanol. After saturation, the different couples of primary antibodies were incubated for 1 h at 37 °C. The PLA probes consisting of secondary antibodies conjugated with complementary oligonucleotides were incubated for 1 h at 37 °C. The amplification step followed the ligation of nucleotides for 100 min at 37 °C. Samples were subsequently analyzed under fluorescence microscopy. For tumor specimen analyses, we used a bright field kit as previously described [5].

### Glutathione transferase (GST) pull-down assay

ERα expression plasmids were transcribed and translated in vitro using T7-coupled reticulocyte lysate in the presence of [^35^S] methionine. GST-fusion proteins were incubated with labeled proteins in 200 µl of binding buffer (Tris 20 mM pH 7.4, NaCl 0.1 M, EDTA 1 mM, glycerol 10%, Igepal 0.25% with 1 mM dithiothreitol and 1% milk) for 2 h at room temperature. After washing, bound proteins were separated by SDS-PAGE and visualized by autoradiography.

### In vitro methylation assays

Immunoprecipitated PRMT1 from MCF-7 cells or GST-PRMT1 fusion protein were incubated with GST-hinge of ERα as described previously [4] in the presence of *S*-adenosyl-l [methyl-^3^H] methionine ([^3^H] SAM 85 Ci/mmol from a 10.4 mM stock solution in dilute HCl/ethanol 9/1 (pH 2.0–2.5); Perkin Elmer) for 1 h at 30 °C. Methylation reactions were quenched by adding Laemmli sample buffer, heated at 95 °C for 5 min, and separated by SDS-PAGE. Following electrophoresis, gels were soaked in Amplify reagent (Sigma) according to the manufacturer’s instructions and visualized by autoradiography. Cold experiments were performed using nonradiolabeled SAM at 0.5 mM. mERα methylation was revealed by western blotting using the anti-methyl-ERα antibody.

### In vitro phosphorylation assays

The assays were performed by incubating the IGF-1R active protein (Merck) with GST-fusion proteins of interest in the presence of adenosine 5′-triphosphate, [ү-^32^PATP] (Perkin Elmer) for 30 min at 30 °C. Phosphorylation reactions were quenched by adding Laemmli buffer, heated at 95 °C for 5 min, and separated by SDS-PAGE. Following electrophoresis, gels were dried and visualized by autoradiography.

### Human breast cancer sample collection

The tumors from 440 patients of the Centre Léon Berard (CLB) with invasive breast cancer, whose clinical and biological data were available from the regularly updated institutional database, were analyzed. Written informed consent was obtained from each patient. The study protocol was approved by the institutional ethics committee. Patient characteristics are presented in Supplementary Table 3.

### Immunohistochemistry staining

Formalin-fixed paraffin-embedded tumor tissues were used for analysis. The pathologist selected representative areas from breast invasive carcinomas. Triplicates from each tumor were inserted into TMA blocks which contained 40 tumors each. After deparaffinization and rehydration, tissue sections were boiled in 10 mM citrate buffer pH 8.0 at 95 °C for 40 min. The slides were then incubated in 5% hydrogen peroxide in sterile water to block the activity of endogenous peroxidases. The slides were then incubated at 37 °C for 1 h with the anti-IGF-1R antibody. The slides were subsequently incubated with a biotinylated secondary antibody bound to a streptavidin peroxidase conjugate (Envision Flex kit Ref: K800021-2, Dako). Bound antibodies were detected by adding the substrate 3,3-diamino benzidine. Sections were counterstained with hematoxylin.

Blinded to the clinical data, IGF-1R expression was evaluated by two observers who assessed both the percentage and the intensity of staining separately. For scoring purposes, the intensity of staining in malignant cells was categorized into four levels (0: no staining, 1: weak staining, 2: moderate staining, 3: strong staining) and the percentage of stained cells was reported separately. Both intensity and percentage scores were then multiplied to conclude a single H score. As IGF-1R expression posed no prognostic value in breast cancer samples (data not shown), the median *H* score (*H* score of 100) was chosen as a cutoff value and the entire cohort was divided into high (>100) and low (**≤**100) IGF-1R-expressing patients. Accordingly (and as only 404 samples were technically assessable after preparation), 202 patients (50%) had low IGF-1 expression and 202 patients (50%) had high IGF-1 expression levels.

### Image acquisition and analysis

The hybridized fluorescent slides were viewed under a Nikon Eclipse Ni microscope. Images were acquired under identical conditions at ×60 magnification. Image acquisition was performed by imaging 4’,6-Diamidino-2-Phenylindole, Dihydrochloride (DAPI) staining at a fixed Z Position while a Z stack of ±5 μm at 1 μm intervals was carried out. The final image was stacked to a single level before further quantification. On each sample, at least 100 cells were counted. Analysis and quantifications of these samples were performed using ImageJ software (free access). PLA dots were quantified on 8-bit images using the “Analyse Particles” command, while cells were counted using the cell counter plugin.

IHC images were also acquired using Nikon Eclipse Ni microscope at ×40 magnification and PLA dots were quantified as described above.

### Statistical analysis

#### Descriptive analysis

The distribution of clinical parameters (cancer subtype, clinical, histological, and immunohistochemical data) was presented as numbers and percentages. Correlations between expression levels and clinical parameters or biomarkers were conducted using Fisher’s exact test. Statistical analyses were carried out using the SPSS v20.0 software (IBN, USA). A statistically significant interaction was considered if the alpha error was less than 5%.

## Supplementary information

Supplemental material 1 to 10 and supp methods

Supplementary Information
