# Supplementary material for: The arginine methyltransferase PRMT1 regulates IGF-1 signaling in breast cancer
Source: Oncogene. 2019 Jan 28;38(21):4015–27. doi: 10.1038/s41388-019-0694-9 (PMC6755991; doi:10.1038/s41388-019-0694-9)
Supplement: Supplementary file 2 — Supplementary Information [file 41388_2019_694_MOESM2_ESM.pdf]

Supplementary Information

The arginine methyltransferase PRMT1 regulates IGF-1 signaling in breast cancer

Ali Choucair, Thuy Ha Pham, Soleilmane Omarjee, Julien Jacquemetton, Loay Kassem, Olivier Trédan, Juliette Rambaud, Elisabetta Marangoni, Laura Corbo, Isabelle Treilleux and Muriel Le Romancer.

Supplementary Figures

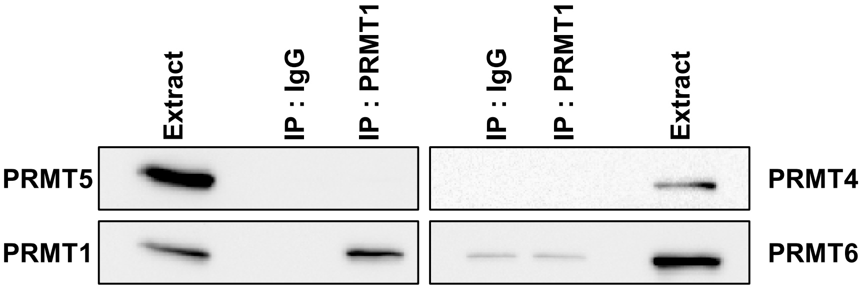

Fig. S1

Supplementary Fig. 1. The anti-PRMT1 antibody immunoprecipitates specifically PRMT1. MCF-7 cell lysates were immunoprecipitated with the anti-PRMT1 antibody. We validated the specificity of this experiment by conducting a Western blot to detect the presence of PRMT1, PRMT4, PRMT5 and PRMT6 using the corresponding antibodies.

Supplementary Figures

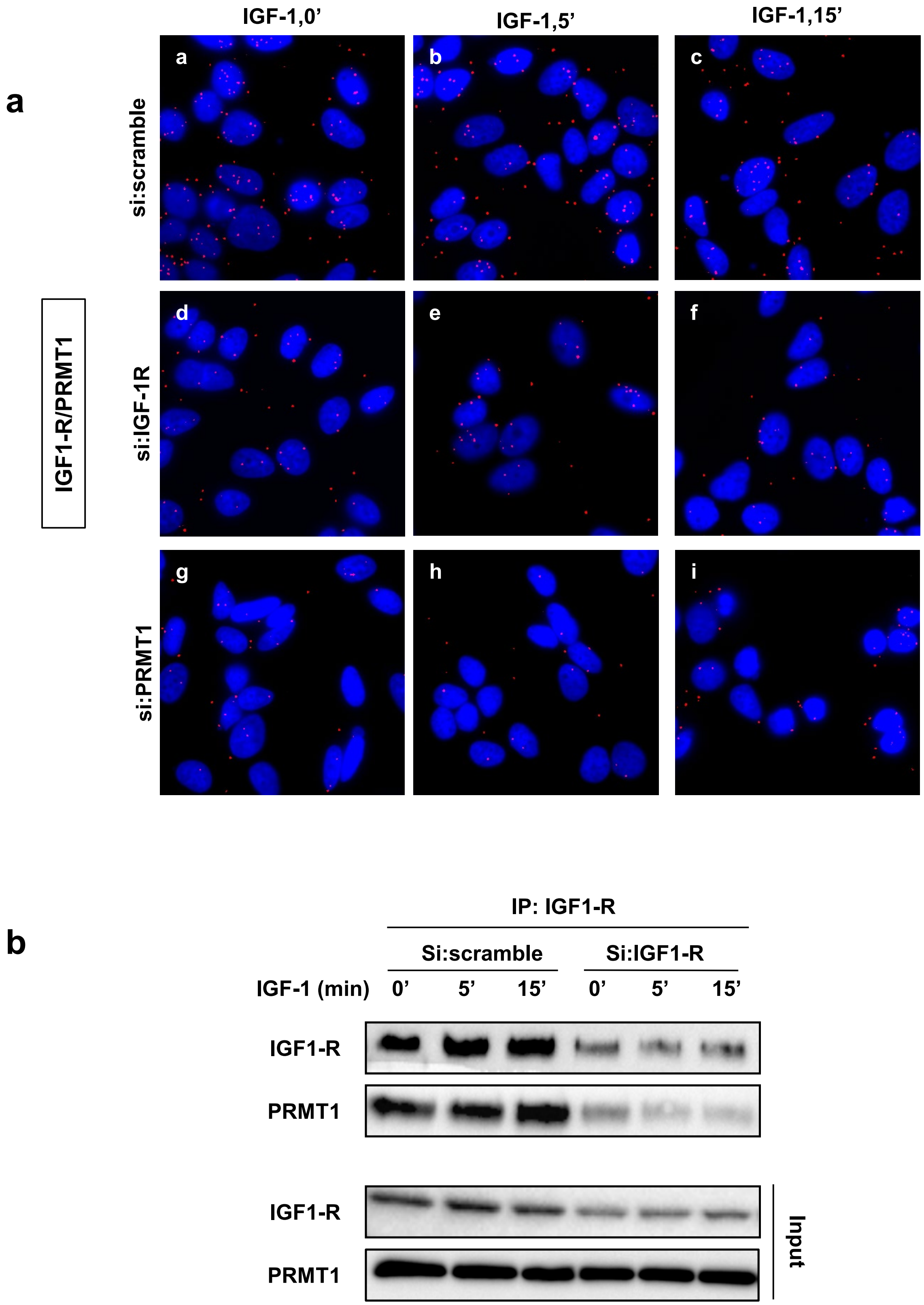

Fig. S2

Supplementary Fig. 2. IGF-1R interacts with PRMT1.

a MCF-7 cells were transfected with si:scramble or siRNAs targeting IGF-1R or PRMT1 for 72 h, then treated with IGF-1 for different periods of time. After fixation, PLA experiments were performed to evaluate IGF-1R/PRMT1 interaction using IGF-1R- and PRMT1-specific antibodies. The detected dimers are represented by red dots. The nuclei were counterstained with mounting medium containing DAPI (blue) (Obj: X60). **b** MCF-7 cells transfected with si:scramble or siRNAs targeting IGF-1R for 72 h, then treated with IGF-1 for different periods of time. Cell extracts were immunoprecipitated with anti-IGF-1R antibody and detected by Western blotting for the presence of PRMT1 and IGF-1R, using the corresponding antibodies. The level of expression of PRMT1 and IGF1-R was also evaluated in the input.

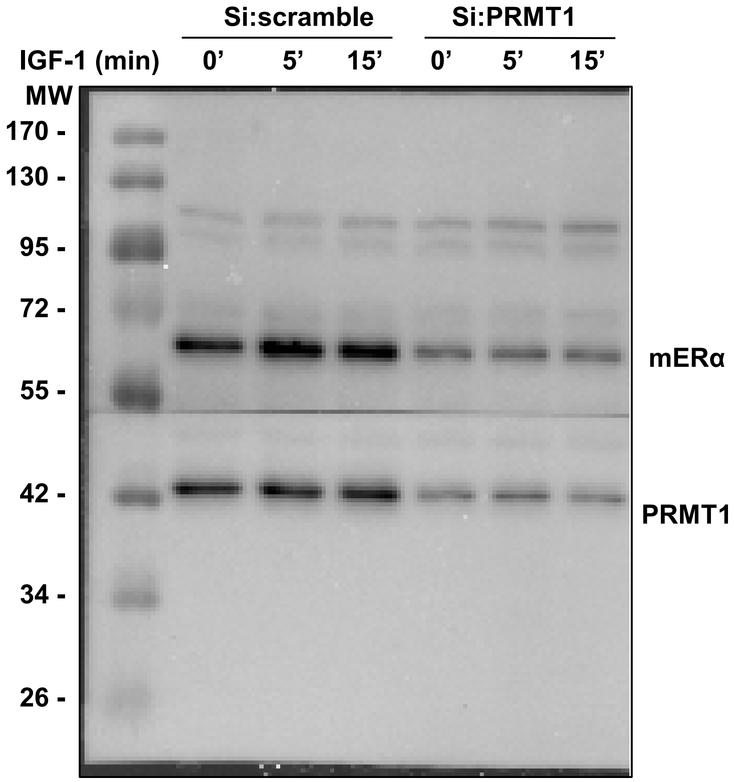
Si:scramble
Si:PRMT1
IGF-1 (min)
0'
5'
15'
0'
5'
15'
MW
170 -
130 -
95 -
72 -
55 -
42 -
34 -
26 -
mERα
PRMT1

Fig. S3

Supplementary Fig. 3. Validation of a new batch of mERα antibody

MCF-7 cells were transfected with si: scramble or a pool of siRNAs targeting PRMT1 for 72 h, and then treated with IGF-1 for different times. The expression of mERα and PRMT1 from Figure 3a is shown as a full panel.

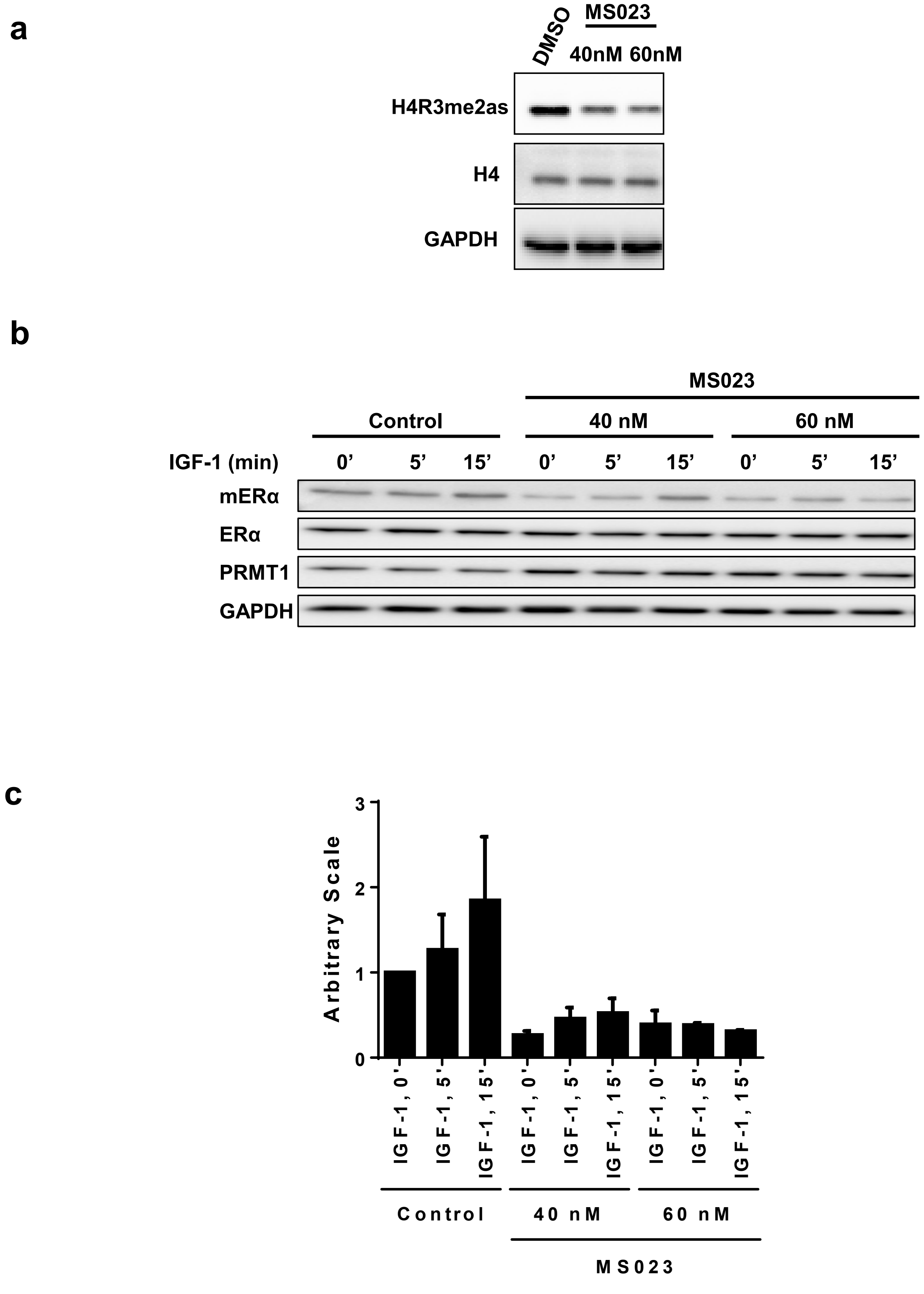
a
DMSO
MS023
40nM 60nM
H4R3me2as
H4
GAPDH
b
MS023
Control
40 nM
60 nM
IGF-1 (min)
0' 5' 15' 0' 5' 15' 0' 5' 15'
mERα
ERα
PRMT1
GAPDH
c
Arbitrary Scale
0
1
2
3
IGF-1, 0'
IGF-1, 5'
IGF-1, 15'
IGF-1, 0'
IGF-1, 5'
IGF-1, 15'
IGF-1, 0'
IGF-1, 5'
IGF-1, 15'
Control
40 nM
60 nM
MS023

Fig. S4

Supplementary Fig. 4. Validation of a PRMT1 inhibitor on its activity.

a MCF-7 cells were treated with or without the PRMT1 inhibitor MS023 (40 and 60 nM) for 48 h. Histones were subsequently acid extracted and the expression of histone H4 and asymmetrical dimethylated histone H4 was assessed by Western blot analysis using specific antibodies. **b** MCF-7 cells were treated with or without the PRMT1 inhibitor MS023 (40 and 60 nM), 48 h before IGF-1 treatment. The expression of ERα methylation, ERα, PRMT1 and GAPDH was then assessed by Western blot analysis. **c** Quantification of the signal from two independent experiments was performed by computer-assisted analysis. Normalization was performed according to ERα expression.

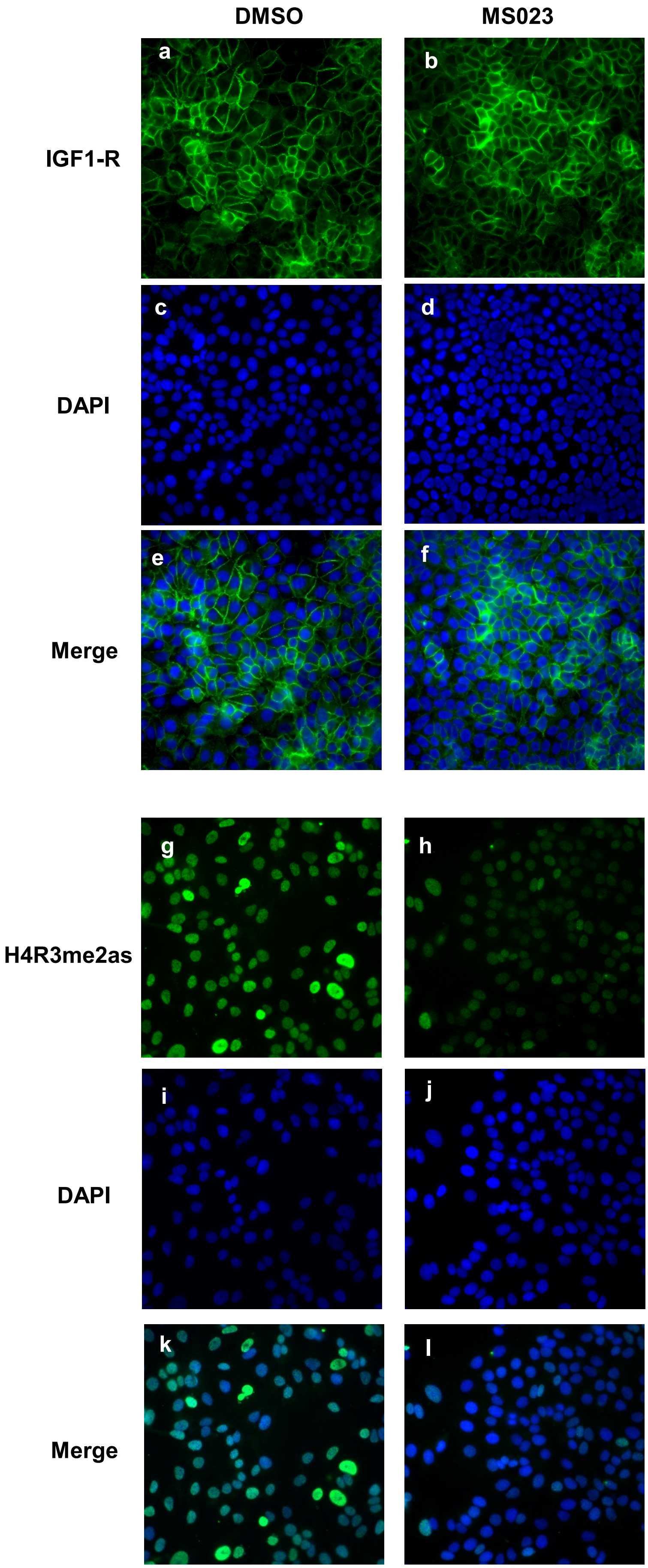
DMSO
MS023
a
b
IGF1-R
c
d
DAPI
e
f
Merge
g
h
H4R3me2as
i
j
DAPI
k
l
Merge

Fig. S5

Supplementary Fig 5. Role of PRMT1 on IGF-1R trafficking.
MCF-7 cells were treated with or without the PRMT1 inhibitor MS023 (60 nM) for 48 h. Methanol-fixed cells were then stained with an anti-IGF-1R antibody. The nuclei were counterstained with mounting medium containing DAPI (blue) (Obj: X60). A positive control was used, namely the asymmetrical dimethylated histone H4 antibody.

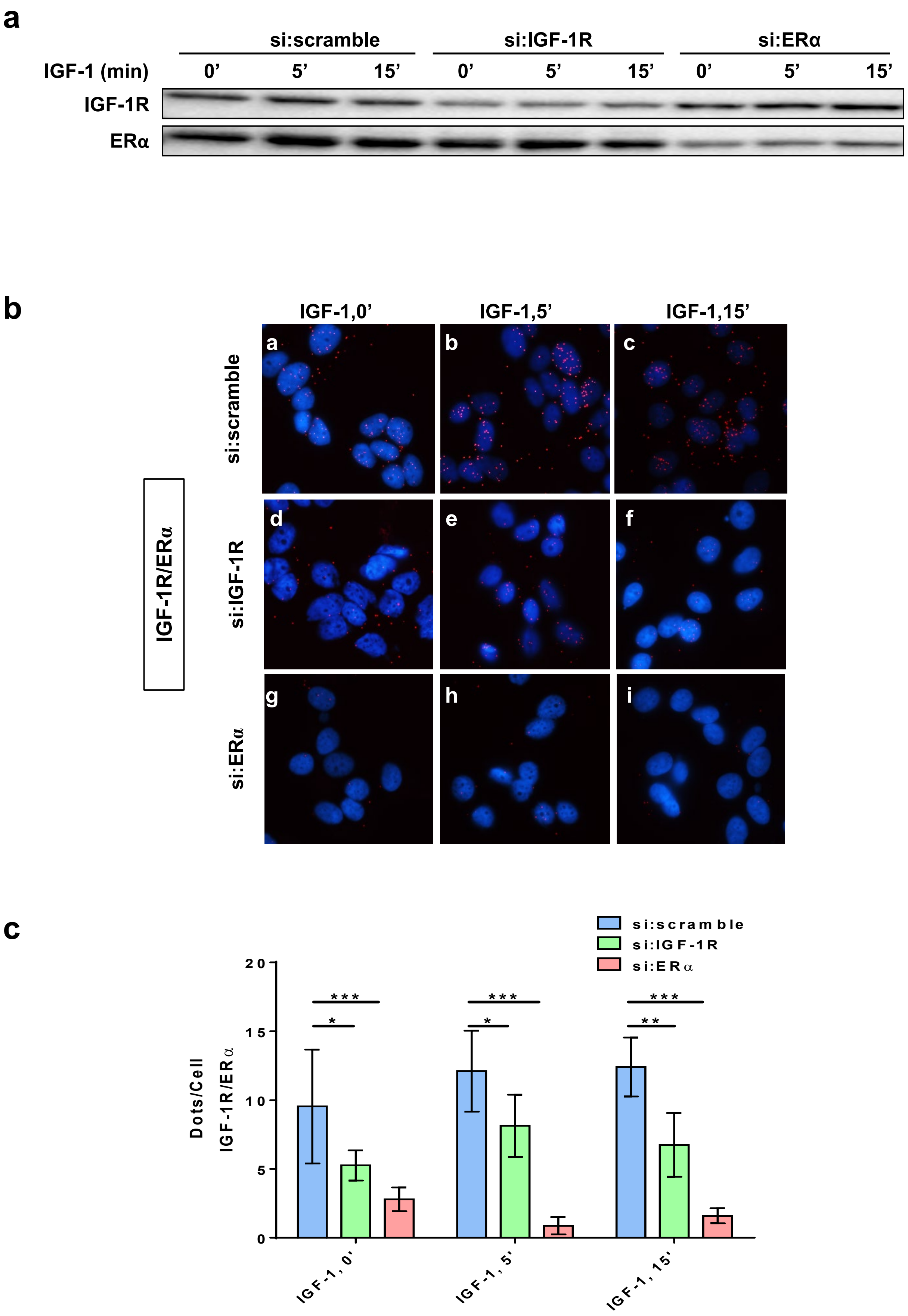

a
si:scramble
si:IGF-1R
si:ERα
IGF-1 (min)
0'
5'
15'
IGF-1R
ERα
b
IGF-1,0'
IGF-1,5'
IGF-1,15'
si:scramble
si:IGF-1R
si:ERα
IGF-1R/ERα
a
b
c
d
e
f
g
h
i
c
si:scramble
si:IGF-1R
si:ERα
Dots/Cell
IGF-1R/ERα
20
15
10
5
0
***
*
**
IGF-1, 0'
IGF-1, 5'
IGF-1, 15'

Fig. S6

Supplementary Fig. 6. Study of IGF-1R/ERα interaction in MCF-7 cells

a MCF-7 cells were transfected with si:scramble or siRNAs targeting IGF-1R or ERα for 72 h, and then treated with IGF-1 for different periods of time. The efficacy of protein inhibition was verified by Western blot analysis using the corresponding antibodies. **b** After siRNA transfection and cell fixation, PLA experiments were performed to evaluate the interactions between IGF-1R/ERα using IGF-1R and ERα specific antibodies. The detected dimers are represented by red dots. The nuclei were counterstained with mounting medium containing DAPI (blue) (Obj: x60). **c** Quantification of the number of dots per cell was performed by computer-assisted analysis as reported in the Materials and Methods section. The mean +/- s.e.m. of one experiment representative of three experiments is shown. The *P*-value was determined using the Student t-test. * $P < 0.05$, *** $P < 0.001$.

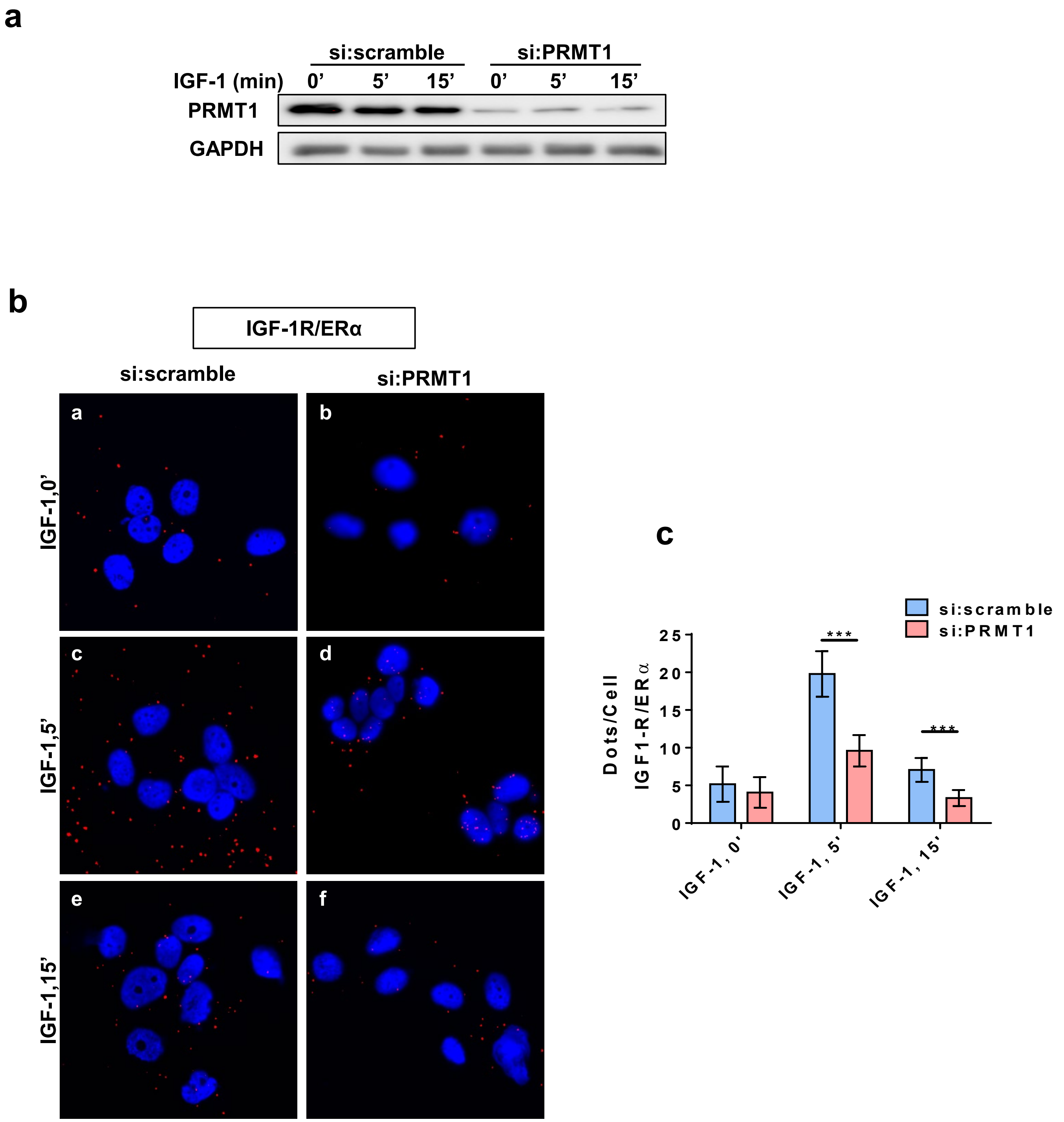
a
si:scramble
si:PRMT1
IGF-1 (min)
0’
5’
15’
0’
5’
15’
PRMT1
GAPDH
b
IGF-1R/ERα
si:scramble
si:PRMT1
a
b
c
d
e
f
IGF-1,0’
IGF-1,5’
IGF-1,15’
c
si:scramble
si:PRMT1
Dots/Cell
IGF1-R/ERα
25
20
15
10
5
0
***
***
IGF-1, 0’
IGF-1, 5’
IGF-1, 15’

Fig. S7

Supplementary Fig. 7. PRMT1 influences IGF-1R/ERα interaction

a MCF-7 cells were transfected with si: scramble or a pool of siRNAs targeting PRMT1 for 72 h, and then treated with IGF-1 for different times. The silencing of PRMT1 was evaluated by Western blot using the PRMT1 antibody and GAPDH expression was used as a loading control. **b** After cell fixation, PLA experiments were carried out to quantify the interactions between IGF-1R/ERα in MCF-7 cells transfected with siRNAs as described in a. **c** Quantification of the number of dots per cell was performed as reported in the Materials and Methods section. The mean +/- s.e.m. of one experiment representative of three experiments is shown. The *P*-value was determined using the Student t-test. *** $P < 0.001$.

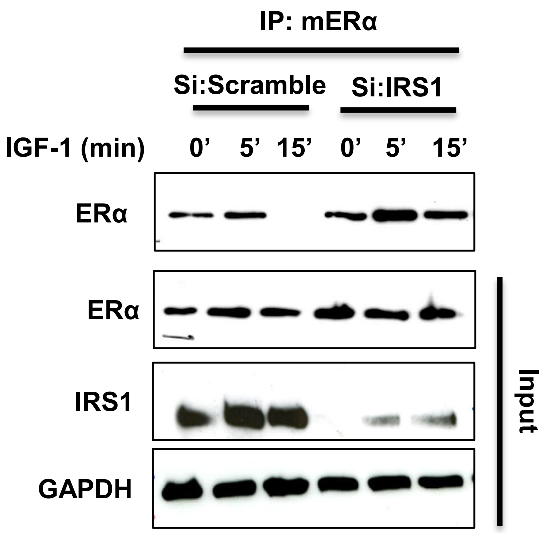
IP: mERα
Si:Scramble
Si:IRS1
IGF-1 (min)
0'
5'
15'
0'
5'
15'
ERα
ERα
IRS1
GAPDH
Input

Fig. S8

Supplementary Fig. 8. Role of IRS1 on ERα methylation.

MCF-7 cells were transfected with si:scramble or siRNAs targeting IRS1 for 72 h, and then treated with IGF-1 for different periods of time. mERα was then immunoprecipitated with the specific antibody followed by Western blotting with anti-ERα antibody. IRS1 ERα and GAPDH expression levels were evaluated in the total cell extracts.

a

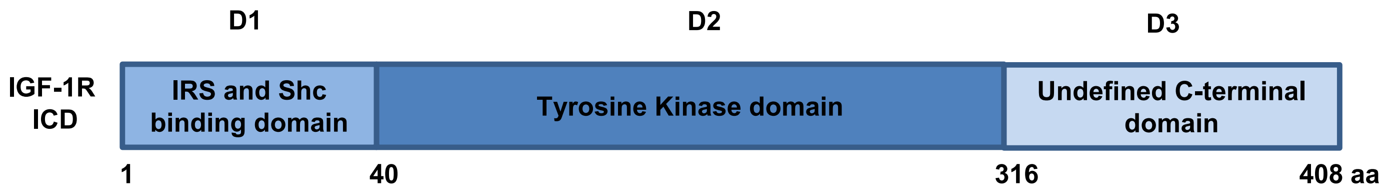
D1
D2
D3
IGF-1R
ICD
IRS and Shc
binding domain
Tyrosine Kinase domain
Undefined C-terminal
domain
1
40
316
408 aa

b

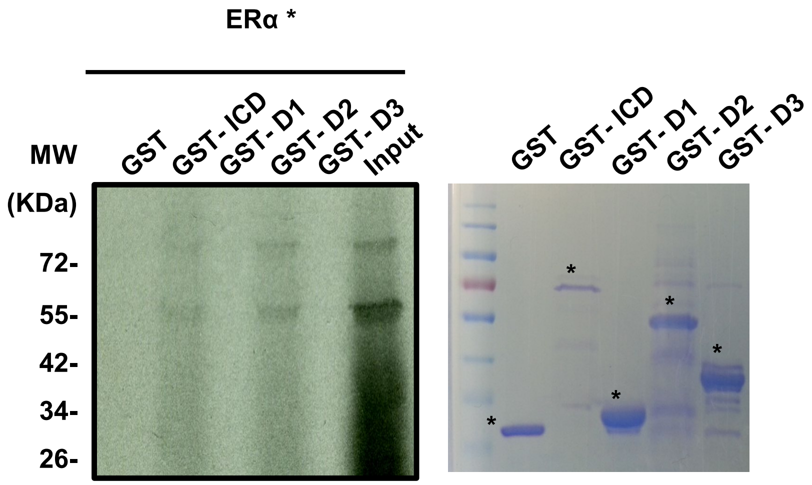
ERα *
MW
(KDa)
GST
GST- ICD
GST- D1
GST- D2
GST- D3
Input
72-
55-
42-
34-
26-
GST
GST- ICD
GST- D1
GST- D2
GST- D3
*

Fig. S9

Supplementary Fig. 9. Interaction between ERα and IGF-1R.
a This figure presents the different structural domains of the intracellular domain (ICD) of IGF-1R. The ICD is divided into 3 domains: the D1 domain contains the IRS1 and Shc binding domain, the D2 domain contains the kinase activity and the D3 domain has an unknown function. **b** GST pull-down assay was performed by incubating *in vitro* ^{35}S-labeled ERα with GST, GST-ICD, GST-D1, GST-D2 and GST-D3 of IGF-1R. The corresponding Coomassie-stained gel is shown in the right-hand panel. * indicates the full length fusion proteins.

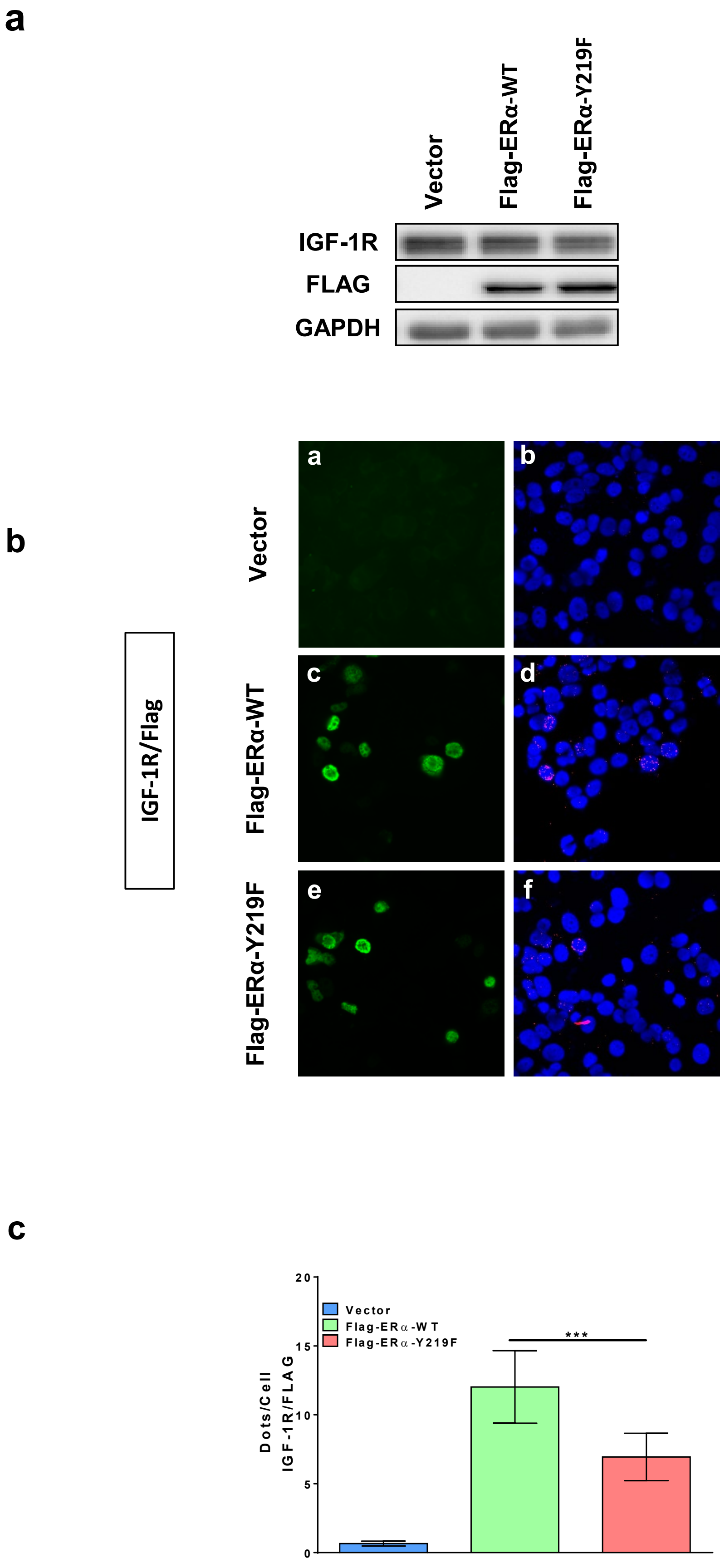
a
Vector
Flag-ERα-WT
Flag-ERα-Y219F
IGF-1R
FLAG
GAPDH
b
IGF-1R/Flag
Vector
Flag-ERα-WT
Flag-ERα-Y219F
a
b
c
d
e
f
c
Dots/Cell
IGF-1R/FLAG
20
15
10
5
0
Vector
Flag-ERα-WT
Flag-ERα-Y219F
***

Fig. S10

Supplementary Fig. 10. ERα phosphorylation regulates ERα binding to IGF-1R.

a MCF-7 cells were transfected with empty pSG5, pSG5-Flag ERα WT or the mutant pSG5-Flag ERα Y219F. The expression of the transfected ERα was assessed by Western blot analysis using the anti-Flag and anti-GAPDH antibodies. **b** PLA experiments were performed to evaluate the interactions between endogenous IGF-1R and transfected ERα using a mouse anti-IGF-1R antibody and a rabbit anti-Flag antibody. The transfected cells were visualized by adding secondary anti-rabbit antibody coupled with Alexa 488 and the nuclei were counterstained with mounting medium containing DAPI (blue) (Obj: X60). **c** Quantification of the number of dots per cell was performed as described in the Materials and Methods section, except that the counting was performed only in transfected cells. The mean +/- s.e.m. of one experiment representative of four experiments is shown. The *P*-value was determined using the Student t-test. *** $P < 0.001$.

Supplementary Table 1. List of the targeted sequences for siRNA experiments

siRNA	Sequence (5'-3')	Reference	Supplier
si:scramble		SR-CL000-005	Eurogentec
si-PRMT1		110970	Ambion
si:ERα	GAAUGUGCCUGGCUAGAGAdTdT		
si:IGF-1R	CUGACUACAGGGAUCUCAUdTdT		

Supplementary Table 2. List and origin of the antibodies used in the current work

Antibody	Company	Ref.	Species	WB	IP	PLA	IHC	IF
Akt	CST	9272	rabbit	1:1000				
ERα-60C	Merck	04-820	rabbit	1:1000				
ERα-HC20	Santa Cruz	sc-543	rabbit			1:500		
Flag	CST	14793	rabbit	1:500		1:2000		
Histone H4	Merck	07-108	rabbit	1:500				
H4R3me2as	Active motif	39705	rabbit	1:500				1:500
IGF-1Rβ	CST	9750	rabbit	1:1000	1:100			1:500
IGF-1Rβ	Santa Cruz	Sc-81167	mouse			1:750		
IGF-1Rβ	CST	3027	rabbit				1:25	
IRS1	CST	3407	rabbit	1:1000				
p-IRS1(Y608/Y612)	Millipore	09-432	mouse	1:1000				
Erk	CST	4695	rabbit	1:2000				
mERα	Home made	2D10	mouse	1:1000	1:100			
p-Akt (Ser473)	CST	9271	rabbit	1:1000				
p-IGF-1Rβ(Y1135)	CST	3918	rabbit	1:1000				
p-Erk	CST	4370	rabbit	1:1000				
P85-PI3K	Millipore	05-212	rabbit	1:1000				
P85-PI3K	Abcam	Ab22653	mouse			1:100		
PRMT1	Millipore	07-404	rabbit			1:500		
PRMT1	Bethyl	A300-722A1	rabbit		1:100			
PRMT1	CST	2449	rabbit	1:1000				
PRMT5	Millipore	07-405	rabbit	1:1000				
PRMT4	CST	3379	rabbit	1:1000				
PRMT6	CST	14641	rabbit	1:1000				
Shc	Millipore	06-203	rabbit	1:1000				
p-Shc (Y239/240)	CST	2434	rabbit	1:1000				
GAPDH	Santa Cruz	Sc-47724	mouse	1:2000				
Src	Santa Cruz	sc-8056	mouse	1:1000		1:100		

Supplementary Table 3. Sample description: distribution of clinical parameters

Clinical parameters were analyzed for the 440 patients included in the TMA study.

Characteristic		Number	percent
**Age group**	*≤ 50 years*	113	26.1%
	>50 years	320	73.9%
**Menopausal status**	*Premenopausal*	121	28.5%
	Post-menopausal	303	71.5%
	Unknown	9	
**BMI**	*≤ 25 kg/m²*	258	61.9%
	> 25 kg/m²	159	38.1%
	Missing	16	
**Tumor size**	*≤2cm*	252	58.2%
	>2cm	181	41.8%
**Axillary LN metastasis**	*No*	184	42.5%
	Yes	249	57.5%
**SBR grade**	*I*	82	18.9%
	II	207	47.8%
	III	144	33.3%
**ERα status**	*Negative*	56	12.9%
	Positive	377	87.1%
**PR status**	*Negative*	109	25.2%
	Positive	324	74.8%
**HER2 status**	*Negative*	397	92.8%
	Positive	31	7.2%
	Missing	5	
**Breast cancer subtype**	*Luminal A*	243	56.1%
	Luminal B	134	30.9%
	HER2 enriched	11	4.6%
	TNBC	45	10.4%
**Adjuvant hormonal regimen**	*Tamoxifen*	173	46.6%
	Tam-AI	198	53.4%
	Missing	62	

Supplementary Materials and Methods

Primers and cloning

The following primers were used to clone the domains of ICD of IGF-1R:

IGF-1R ICD D1 Fwd: 5’ CATAGAAAGAGAAATAACAGCAGG 3’
IGF-1R ICD D1 Rev: 5’ CTCGAGCTTCTCCCGAGCCACCTCCC 3’

IGF-1R ICD D2 Fwd: 5’ GGATCCATCACCATGAGCCGGGAACTTG 3’

IGF-1R ICD D2 Rev: 5’ CTCGAGGAAGCCAGGCTCCATCTCCTC 3’

IGF-1R ICD D3 Fwd: 5’ GGATCCCGGGAGGTCTCCTTCTACTACAG 3’
IGF-1R ICD D3 Rev: 5’ CTCGAGTCAGCAGGTCGAAGACTGGGGC 3’
The primer sequences in bold were used to clone the full ICD of IGF-1R.

ICD and its fragments were cloned into the pGEX4T1 vector in BamH1/Xho1 sites in phase with the GST. The constructs were verified by sequencing.

Immunofluorescence

MCF-7 cells ($7x10^4$) were grown on coverslips in 12-well plates. After treatment, cells were fixed in methanol for 2 min and washed twice in PBS. Non-specific binding was blocked using a 1% gelatin solution for 30 min at room temperature and cells were incubated with primary antibodies for 1 h at 37°C. After PBS washes, the cells were incubated for another 1 h at 37°C with the secondary antibody Alexa Fluor 488 (Molecular Probes) in Dako diluent, then washed in PBS and mounted on glass slides in mounting solution (Dako). The images were acquired using a fluorescent microscope.

Histone extraction

80 µl of Buffer (20mM Tris-HCl with pH 8.8 and 20mM $CaCl_2$) was added into ($5x10^6$) cells. The suspension was mixed well before 9ul of 3% SDS and 10% β-mercaptoethanol (100ul β-mercaptoethanol, 300 µl SDS and 600 µl H_2O) were loaded. The mixture then was boiled at

95ºC in water bath for 2min then cooled in ice. After that, 5 μl of DNAse solution along with 5 μl of RNAse solution in Buffer 2 (500 mM Tris-HCl with pH 7.0 and 50 mM $MgCl_2$) were applied. The sample then was mixed well for being homogenous, followed by being boiled with Laemli 1X for 5 min and analyzed by Western blotting.
